# Short-range spin-phonon coupling in in-plane CuO nanowires: a low-temperature Raman investigation

**DOI:** 10.1186/1556-276X-8-398

**Published:** 2013-09-25

**Authors:** Po-Hsun Shih, Chia-Liang Cheng, Sheng Yun Wu

**Affiliations:** 1 Department of Physics, National Dong Hwa University, Hualien 97401, Taiwan

**Keywords:** Raman spectroscopy, Cupric oxide, Nanowire, Spin-phonon coupling

## Abstract

We report an application of low-temperature Raman scattering on in-plane CuO nanowires, in which an overview of the characteristic parameter of spin-phonon coefficient, the interaction of incident light with the spin degrees of freedom, and size effects will be given. The appearance of spin-phonon coefficient decrease reflects the existence of finite size effect.

## Background

Low-dimensional nanosized effects in CuO systems, especially their different physical properties such as spin-spin [1,2], electron–phonon [3], spin-phonon interactions [4], and giant negative thermal expansion have recently received a lot of attention [5]. The spin-spin superexchange interaction occurs via the oxygen orbital [4,6]. The magnetic interactions and Néel transition temperature (*T*_N_) of the CuO system are strongly dependent on the exchange interaction and the number of neighboring atoms. A transition from a first-order transition to a commensurate antiferromagnetic state near *T*_N_ ~ 213 K reported for bulk CuO from neutron scattering experiments [7,8] is well understood. Controlling the size of CuO nanocrystals resulted in short-range correlation and commensurate antiferromagnetic (AFM) ordering, where the *T*_N_ decreased from the bulk value of 213 K [9-11], with decreasing particle size, down to 40 K for 6.6-nm nanoparticles [1,2] and 13 K for 2- to 3-nm nanorods [12]. It is known that spin-phonon coupling is usually weak and undetectable because symmetric vibrations of relevant atoms will cancel the contributions from negative and positive displacements. The main feature of cupric oxide is the low-symmetry monoclinic lattice, which differs from the other transition metal monoxides, e.g., MnO, FeO, CoO, and NiO with rock salt structure [13]. The low symmetry of the CuO lattice and the anisotropic dispersion curves indicated lattice vibration which caused a modulation of the spin-phonon interaction. This originated from slight changes in the inter-ionic distances and bond angles, leading to spin-phonon coupling that can be detected in the Raman spectrum, to produce a weak feature at about 230 cm^−1^ below *T*_N_[14,15]. The discovery of spin-phonon coupling in CuO nanocrystals has led to renewed interest in this phenomenon. Up to now, there have been few experimental alternatives for the determination of the size effect of spin-phonon coupling of CuO nanowires. In this study, low-temperature Raman spectroscopy is employed to investigate the size effects of spin-phonon coupling in in-plane CuO nanowires. Low-temperature Raman spectroscopy has the high spatial resolution and sensitivity necessary for probing the local atomic vibrations of nanowires. Our results reveal that below Néel temperature there is a ready shift of the spin-phonon coefficient *λ*_sp_ decreases as the mean diameter of in-plane CuO nanowire decreases, exhibiting a long- to short-range spin-phonon coupling that can be nicely described with the expected theoretical order parameter as due to antiferromagnetic ordering in in-plane CuO nanowires.

## Methods

**Figure 1:**
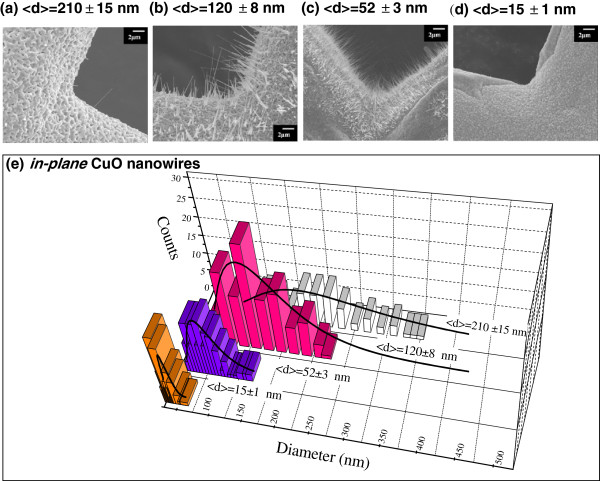
**Morphology of the in-plane CuO nanowires.** SEM images of the in-plane CuO nanowires synthesized at various temperatures **(a, b, c, d)**. The distributions of the mean diameter of the nanowires obtained from a portion of the SEM image **(e)**. The solid lines represent the fitting curves assuming the log-normal function, where <*d*> is the mean diameter of the nanowires.

A series of in-plane CuO nanowires with various diameters were fabricated. The samples were prepared by a process where a pure copper grid was placed in a ceramic boat inside a quartz tube, which was then evacuated to about 10^−3^ Torr using a mechanical pump. They were then heated in a tube furnace at about 200°C for 2 h for degassing, after which the samples were heated to various temperatures ranging from 300°C to 600°C for 2 h under mixed argon (100 sccm) and oxygen (10 sccm) gas. Details of specimen preparation and characterization have been described in a previous paper [16]. Transmission electron microscopy (TEM) and high-resolution transmission microscopy (HRTEM) images from a JEM-3010 transmission electron microscope (JEOL Ltd., Tokyo, Japan) were obtained to study the crystalline structure. The results of an early study show that the prepared nanowires are crystalline [16], revealing a monoclinic unique Y structure with lattice parameters of *a* = 4.63 Å, *b* = 3.55 Å, *c* = 5.16 Å, and *β* = 99°52′. The morphology of the prepared nanowires was characterized using field-emission scanning electron microscopy (FESEM; JEOL JSM-6500 F). The SEM images in Figure 1a,b,c,d show the morphology of the CuO nanowires with various diameters which were synthesized at *T* = 600°C, 500°C, 400°C, and 300°C, respectively. It can be seen that the in-plane CuO grew homogeneously on the copper grid substrate to form straight nanowires. Observation of uniform nanowires (with lateral dimensions in the nanoscale order of tens to hundreds nanometers) shows that they grew up to a few microns in length. Figure 1e shows that the distribution of the nanowires was quite asymmetric. The solid lines represent the fitting curves assuming the log-normal function^a^. The mean diameters obtained from the fits of log-normal distribution are <*d*> = 210 ± 15 nm, 120 ± 8 nm, 52 ± 3 nm, and 15 ± 1 nm, respectively. The value obtained for the standard deviation of the distribution profile *σ* reveals that the increase with broadening was presumably due to the crystalline effects.

## Results and discussion

**Figure 2:**
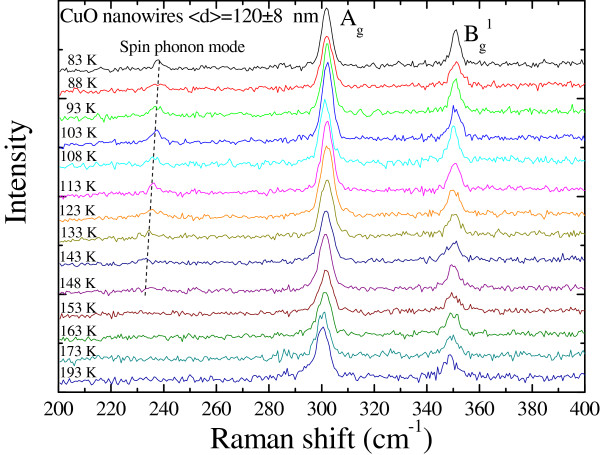
**Series of Raman spectra taken at various temperatures of CuO nanowires with a mean average diameter <*d*> = 120 ± 8 nm.** Two main phonon modes corresponding to the *A*_g_ and *B*_g_^1^ symmetries, respectively, are revealed. As the temperature was reduced to143 K, a well-defined peak at 238 cm^−1^ developed, signifying the spin-phonon coupling.

All low-temperature Raman spectra were measured using a Jobin Yvon 64000 Raman microscope (HORIBA, Minami-ku, Kyoto, Japan) equipped with a Linkam optical DSC system (THMS600; Linkam Scientific Instruments, Surrey, UK). The results were utilized to investigate the spectroscopic properties of CuO nanowire at various temperatures. The specimens were mounted on a non-background sample holder fixed to a cold head in a high-vacuum (<10^−3^ Torr), low-temperature (approximately 80 K) chamber. The CuO nanowire was excited by focusing a 514.5-nm Ar ion laser (Coherent Inc., Santa Clara, CA, USA) with a 5-mW laser power on the sample to form a spot size of approximately 1 μm in diameter, giving a power density of 10^2^ W/cm^2^. From the factor group analysis of the zone center modes for the monoclinic structure, given by Rousseau et al. [17], there are three Raman active modes (*A*_g_, *B*_g_^1^, and *B*_g_^2^) predicted in the spectra of CuO nanowires. Figure 2 shows an example of a series of Raman spectra taken at various temperatures, covering the antiferromagnetic transition temperature, with a mean diameter of 120 ± 8 nm. There are two phonon modes revealed in the Raman spectra taken of the CuO nanowires at *T* = 193 K at 300.2 and 348.8 cm^−1^[18], which are related to *A*_g_ and *B*_g_^1^ symmetries [19,20]. The peak position is lower than the value of the bulk CuO (*A*_g_ = 301 cm^−1^ and *B*_g_^1^ = 348 cm^−1^) [21], reflecting the size effect which acts to confine the lattice vibration in the radial directions resulting in a shift in the *A*_g_ and *B*_g_^1^ symmetries. As the temperature decreases to 83 K, it can be clearly seen that the peak positions of the *A*_g_ and *B*_g_^1^ modes around 301.8 and 350.9 cm^−1^, shown at the top of Figure 2, shifted toward higher Raman frequencies. While the temperature increased from 83 to 193 K, the peak position of the *A*_g_ mode softened by 0.7%. Since the frequency of the phonon mode is related to Cu-O stretching, it is expected that the frequency will downshift with increasing temperature, primarily due to the softening of the force constants that originate from the thermal expansion of the Cu-O bonds, resulting from the change in vibrational amplitude [22,23]. In the study, the high resolution of our spectrometer allowed detection of relative change as small as 0.5 cm^−1^, and the vibrational frequency of a phonon mode can be used to determine the spin-phonon interaction. A phonon-phonon effect originates from the dynamical motion of lattice displacements, which are strongly coupled to the spin degrees of freedom dynamically below the magnetic ordering temperature. This coupling between the lattice and the spin degrees of freedom is named as spin-phonon. As shown in Figure 2, with decreasing temperature, a well-defined peak developed at 231 cm^−1^ signifying the spin-phonon coupling [8,19] which shows that a noticeable shift to lower frequency is sensitive to the temperature variation.

**Figure 3:**
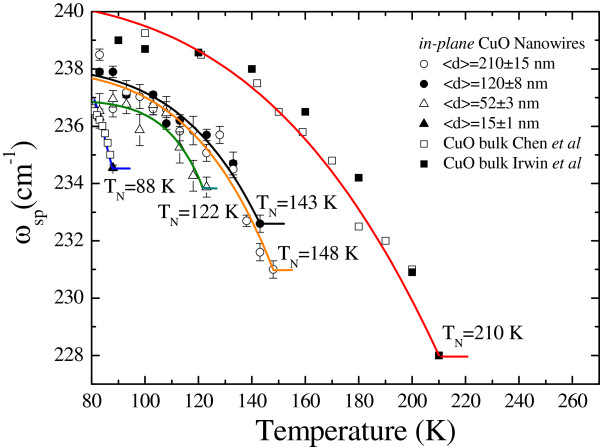
**Temperature variations of the spin-phonon modes of CuO nanowires with various mean diameters.** The solid line represents the fit by the ordering parameter.

**Figure 4:**
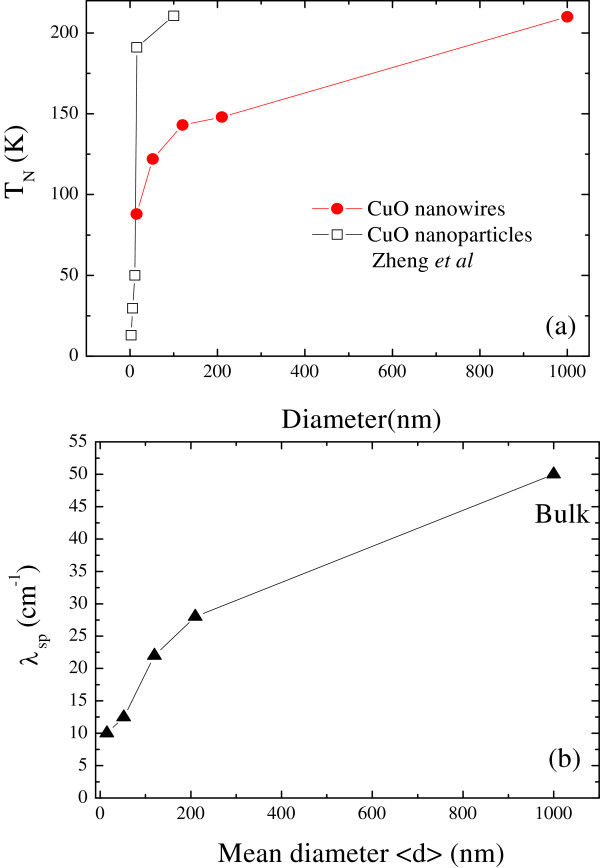
**Size effects of Néel temperature and spin-phonon coupling coefficients.** The obtained Néel temperature **(a)** and spin-phonon coupling coefficients **(b)** as a function of mean diameter, which showed a tendency to decrease with reduction in diameter.

Figure 3 shows the temperature dependence of the spin-phonon mode for in-plane CuO nanowires of various diameters. Typical examples for bulk CuO are shown in Figure 3, indicated by open and solid squares [8]. It has been suggested in previous reports that the temperature dependence of the spin-phonon mode (the origin of the peak at 228 cm^−1^) might be associated with magnetic ordering, the frequency shift corresponding to the spin-correlation function times a spin-phonon coupling coefficient *λ*_sp_. The temperature dependence of the spin-phonon peak can be represented as $\omega_{\mathrm{sp}}=\omega_{\mathrm{sp}}^{o}+\frac{1}{4}\lambda_{\mathrm{sp}}\phi \left(T\right)$, where $\omega_{\mathrm{sp}}^{o}$ is the Raman shift in the absence of spin-phonon coupling at *T*_N_ and *ϕ*(*T*) is the order parameter estimated from the mean field theory [24]. The order parameter can be described as *ϕ*(*T*) = 1 − (*T*/*T*_*N*_)^*γ*^, where the order parameter γ varied from 3.4 ± 0.2 to 20 ± 5. The solid curves indicate the theoretical fitting, and the corresponding parameters are presented in Table 1. The size effect acts to confine the spin-phonon coupling by increasing the *T*_N_ from 210 to 88 K, as shown in Figure 4a, when the size is reduced from bulk to 15 ± 1 nm (see for comparison *T*_N_ = 213 K for CuO single crystal and powder [8,16]). The obtained spin-phonon coupling coefficient *λ*_sp_ also tends to decrease with decreased phonon amplitudes as the diameter decreased, as shown in Figure 4b, revealing the existence of short-range coupling. This result is consistent with past reports which state that the magnetic transition temperature of Cr_2_O_3_[25,26] and CuO nanoparticles (open square) is reduced [12], which can be attributed to the fact that the ground state fails to develop long-range antiferromagnetic ordering. This occurs because of quantum lattice fluctuations and being energetically favorable to some kinds of short-range order state, resulting in a lower spin-phonon coefficient with reduced size [27,28]. The magnitudes of these obtained *λ*_sp_ values are intermediate compared to approximately 1 cm^−1^ for FeF_2_ and MnF_2_[24], and approximately 50 cm^−1^ for bulk CuO [8], indicating that the size effects could result in a tendency to weaken the strong spin-phonon coupling. A minimum spin-phonon coefficient of *λ*_sp_ = 10 cm^−1^ was obtained in <*d*> = 15 ± 1 nm in-plane CuO nanowires, which was found to be weaker by a factor of 0.018 than the nearest neighbor spin-spin coupling strength of *J* = 552 cm^−1^ for one-dimensional antiferromagnetic Heisenberg chain [29]. In general, the spin-orbit interaction will induce a small orbital moment, which couples the magnetic moment to crystalline axes of the phonon vibration. Anharmonic effects are expected and caused the phonon and spin contribution to mix because the *λ*_sp_ decreases as the diameter of the CuO nanowires decreases.

**Table 1:** Summary of the fitting results of the in-plane CuO nanowires

**Size (nm)**	***T***_**N**_**(K)**	$\omega_{\text{sp}}^{o}$ **(cm**^**−1**^**)**	***λ***_**sp**_**(cm**^**−1**^**)**	***γ***
Bulk^a^	210	228	50	3.4 ± 0.2
210 ± 15	148	231	28	4.5 ± 0.5
120 ± 8	143	232.6	22	5.1 ± 0.2
52 ± 3	122	233.8	12.48	8 ± 1
15 ± 1	88	234.5	10	20 ± 5

^a^From [8,15].

## Conclusions

In conclusion, we investigate the size dependence of CuO nanowires and the nanosized spin-phonon effects. Raising the temperature and decreasing the diameter of CuO nanowires result in the weakening of spin-phonon coupling. The temperature variations of the spin-phonon mode at various diameters are in good agreement with the theoretical results. We found that the spin-phonon mode varies with the size of the CuO nanowires and in corroboration with the strength of spin-phonon coupling. Our result reveals that low-temperature Raman scattering techniques are a useful tool to probe the short-range spin-phonon coupling and exchange energy between antiferromagnetic next-nearest neighboring magnons in nanocrystals below the Néel temperature. The application of low-temperature Raman spectroscopy on magnetic nanostructures represents an extremely active and exciting field for the benefit of science and technology at the nanoscale. The rising new phenomena and technical possibilities open new avenues in the characterization of short-range spin-phonon interactions but also for the understanding of the fundamental process of magnetic correlation growth in nanomaterials.

## Endnote

^a^ The log-normal distribution is defined as follows: $f\left(d\right)=\frac{1}{\mathrm{d\sigma}\sqrt{2\pi }}\exp\left(-\frac{{\left(\mathrm{lnd}‒\ln<d>\right)}^{2}}{2\sigma^{2}}\right)$, where <*d*> is the mean value and *σ* is the standard deviation of the function.

## Competing interests

The authors declare that they have no competing interests.

## Authors’ contributions

SYW wrote, conceived of, and designed the experiments. PHS grew the samples and analyzed the data. CLC contributed the Raman experimental facility and valuable discussions. All authors discussed the results, contributed to the manuscript text, commented on the manuscript, and approved its final version.

## References

[B1] Punnoose A Magnone H Seehra MS Bonevich J Bulk to nanoscale magnetism and exchange bias in CuO nanoparticles Phys Rev B 2001 8 174420

[B2] Seehra MS Punnoose A Particle size dependence of exchange-bias and coercivity in CuO nanoparticles Solid State Commun 2003 8 299 302 10.1016/j.ssc.2003.08.029

[B3] Fan H Zou B Liu Y Xie S Size effect on the electron–phonon coupling in CuO nanocrystals Nanotechnology 2006 8 1099 10.1088/0957-4484/17/4/042 21727387

[B4] Tajiri S Inoue J-I Ferromagnetic-antiferromagnetic transition in (La-*R*)_4_Ba_2_Cu_2_O_10_ Phys Rev B 2006 8 092411

[B5] Zheng XG Kubozono H Yamada H Kato K Ishiwata Y Xu CN Giant negative thermal expansion in magnetic nanocrystals Nat Nanotechnol 2008 8 724 726 10.1038/nnano.2008.309 19057591

[B6] Shimizu T Matsumoto T Goto A Chandrasekhar Rao TV Yoshimura K Kosuge K Spin susceptibility and superexchange interaction in the antiferromagnet CuO Phys Rev B 2003 8 224433

[B7] Yang BX Thurston TR Tranquada JM Shirane G Magnetic neutron scattering study of single-crystal cupric oxide Phys Rev B 1989 8 4343 4349 10.1103/PhysRevB.39.4343 9948776

[B8] Chen XK Irwin JC Franck JF Evidence for a strong spin-phonon interaction in cupric oxide Phys Rev B 1995 8 R13130 R13133 10.1103/PhysRevB.52.R13130 9980496

[B9] Forsyth JB Brown PJ Wanklyn BM Magnetism in cupric oxide J Phys C 1998 8 2917

[B10] Brown PJ Chattopadhyay T Forsyth JB Nunez V Antiferromagnetism in CuO studied by neutron polarimetry J Phys Condens Matter 1991 8 4281 10.1088/0953-8984/3/23/016

[B11] Yang BX Tranquada JM Shirane G Neutron scattering studies of the magnetic structure of cupric oxide Phys Rev B 1988 8 174 178 10.1103/PhysRevB.38.174 9945174

[B12] Zheng XG Xu CN Nishikubo K Nishiyama K Higemoto W Moon WJ Tanaka E Otabe ES Finite size effects on Néel temperature in antiferromagnetic nanoparticles Phys Rev B 2005 8 014464

[B13] White RM Geballe TH Long Range Order in Solids 1979 New York: Academic

[B14] Chrzanowski J Irwin JC Raman scattering from cupric oxide Solid State Commun 1989 8 11 14 10.1016/0038-1098(89)90457-2

[B15] Irwin JC Chrzanowski J Wei T Lockwood DJ Wold A Raman scattering from single crystals of cupric oxide Physica C 1990 8 456 464 10.1016/0921-4534(90)90044-F

[B16] Cheng C-L Ma Y-R Chou MH Huang CY Yeh V Wu SY Direct observation of short-circuit diffusion during the formation of a single cupric oxide nanowire Nanotechnology 2007 8 245604 10.1088/0957-4484/18/24/245604

[B17] Rousseau DL Bauman RP Porto SPS Normal mode determination in crystals J Raman Spectrosc 1981 8 253 290 10.1002/jrs.1250100152

[B18] Hagemann H Bill H Sadowski W Walker E Francçis M Raman spectra of single crystal CuO Solid State Commun 1990 8 447 451 10.1016/0038-1098(90)90048-G

[B19] Goldstein HF Kim DS Yu PY Bourne LC Raman study of CuO single crystals Phys Rev B 1990 8 7192 7194 10.1103/PhysRevB.41.7192 9992981

[B20] Campbell IH Fauchet PM The effects of microcrystal size and shape on the one phonon Raman spectra of crystalline semiconductors Solid State Commun 1986 8 739 741 10.1016/0038-1098(86)90513-2

[B21] Kliche G Popovic ZV Far-infrared spectroscopic investigations on CuO Phys Rev B 1990 8 10060 10066 10.1103/PhysRevB.42.10060 9995261

[B22] Xu JF Ji W Shen ZX Li WS Tang SH Ye XR Jia DZ Xin XQ Raman spectra of CuO nanocrystals J Raman Spectrosc 1999 8 413 415 10.1002/(SICI)1097-4555(199905)30:5<413::AID-JRS387>3.0.CO;2-N

[B23] Balkanski M Wallis RF Haro E Anharmonic effects in light scattering due to optical phonons in silicon Phys Rev B 1983 8 1928 1934 10.1103/PhysRevB.28.1928

[B24] Lockwood DJ Gottam MG The spin‒phonon interaction in FeF_2_ and MnF_2_studied by Raman spectroscopy J Appl Phys 1988 8 5876 10.1063/1.342186

[B25] Tobia D Winker E Zysler RD Granada M Troiani HE Size dependence of the magnetic properties of antiferromagnetic Cr_2_O_3_ nanoparticles Phys Rev B 2008 8 104412

[B26] Hung CH Shih PH Wu FY Li WH Wu SY Chan TS Sheu HS Spin-phonon coupling effects in antiferromagnetic Cr_2_O_3_ nanoparticles J Nanosci Nanotechnol 2010 8 4596 4601 10.1166/jnn.2010.1703 21128463

[B27] Iliev MN Guo H Gupta A Raman spectroscopy evidence of strong spin-phonon coupling in epitaxial thin films of the double perovskite La_2_NiMnO_6_ Appl Phys Lett 2007 8 151914 10.1063/1.2721142

[B28] Zheng H Quantum lattice fluctuations as a source of frustration in the antiferromagnetic Heisenberg model on a square lattice Phys Lett A 1995 8 409 415 10.1016/0375-9601(95)00086-I

[B29] Bonner JC Fisher ME Linear magnetic chains with anisotropic coupling Phys Rev 1964 8 A640 A658 10.1103/PhysRev.135.A640

